# Relationship between motor performance and cortical activity of older neurological disorder patients with dyskinesia using fNIRS: A systematic review

**DOI:** 10.3389/fphys.2023.1153469

**Published:** 2023-03-27

**Authors:** Yunzhi Wu, Yuqi Dong, Yunqi Tang, Weiran Wang, Yulong Bo, Cui Zhang

**Affiliations:** ^1^ Graduate School, Shandong Sport University, Jinan, Shandong, China; ^2^ College of Art and Design, Shaanxi University of Science and Technology, Xi’an, Shaanxi, China; ^3^ Laboratory of Sports Biomechanics, Shandong Institute of Sport Science, Jinan, Shandong, China

**Keywords:** Parkinson, stroke, multiple sclerosis, gait, balance, postural control

## Abstract

**Background:** Neurological disorders with dyskinesia would seriously affect older people’s daily activities, which is not only associated with the degeneration or injury of the musculoskeletal or the nervous system but also associated with complex linkage between them. This study aims to review the relationship between motor performance and cortical activity of typical older neurological disorder patients with dyskinesia during walking and balance tasks.

**Methods:** Scopus, PubMed, and Web of Science databases were searched. Articles that described gait or balance performance and cortical activity of older Parkinson’s disease (PD), multiple sclerosis, and stroke patients using functional near-infrared spectroscopy were screened by the reviewers. A total of 23 full-text articles were included for review, following an initial yield of 377 studies.

**Results:** Participants were mostly PD patients, the prefrontal cortex was the favorite region of interest, and walking was the most popular test motor task, interventional studies were four. Seven studies used statistical methods to interpret the relationship between motor performance and cortical activation. The motor performance and cortical activation were simultaneously affected under difficult walking and balance task conditions. The concurrent changes of motor performance and cortical activation in reviewed studies contained the same direction change and different direction change.

**Conclusion:** Most of the reviewed studies reported poor motor performance and increased cortical activation of PD, stroke and multiple sclerosis older patients. The external motor performance such as step speed were analyzed only. The design and results were not comprehensive and profound. More than 5 weeks walking training or physiotherapy can contribute to motor function promotion as well as cortices activation of PD and stroke patients. Thus, further study is needed for more statistical analysis on the relationship between motor performance and activation of the motor-related cortex. More different type and program sports training intervention studies are needed to perform.

## 1 Introduction

Neurological disorders encompass diseases of the brain and nervous system are the leading cause of disability (Murray et al., 2013) and contribute to 3% of disability-adjusted life years (Murray et al., 2012; Caliandro et al., 2020). Cerebrovascular injury (51%), neuromuscular disorders (7%), cognitive disorders (25%), and central nervous system infections (0.6%) are the common symptoms of older patients with neurological disorders (Bacellar et al., 2017), which would lead to dyskinesia (Defebvre and Krystkowiak, 2016; Harmon et al., 2019; Reich and Savitt, 2019). Dyskinesia would seriously affect older peoples’ daily activities, especially walking and balance disability (Osoba et al., 2019). The expensive healthcare costs and additional neurology resource needs of longitudinal intervention burden the family and society. Parkinson’s disease (PD), multiple sclerosis (MS), and stroke are common age-related neurological diseases interrelated with dyskinesia (Bonilauri et al., 2020).

The abnormal motor performance of stroke, PD, and MS patients, such as the step speed, step length, step width, step frequency, gait variability, stance time has been investigated (Hausdorff et al., 2007; Nutt et al., 2011; Socie and Sosnoff, 2013; Chisholm et al., 2014; Maidan et al., 2015; Belluscio et al., 2019). However, the investigation was not enough. Dyskinesia means impairment of control over ordinary muscle movement, which is not only associated with the degeneration or injury of the musculoskeletal or the nervous system but also associated with the complex linkage between them. Motor performance is one of the external representation of the musculoskeletal system, and brain cortical activity is one of the external representation of the central nervous system. If the changes and relationship of motor performance and cortical activity can be analyzed during the patients’ movement, it would help probe the mechanism of dyskinesia and efficient rehabilitation methods for neurological disorder patients. However, testing the cortical activity of the brain during actual human movement is difficult.

The recent advancement in technologies such as Functional near-infrared spectroscopy (fNIRS), and portable electroencephalography has allowed for the investigation of brain function during realtime human movements in the natural environment freely. fNIRS is a non-invasive, repeatable, and reliable functional neuroimaging technology based on the theory of neurovascular coupling and optical spectroscopy (Villringer and Chance, 1997; Leff et al., 2011). An increase in neural activity of brain causes an increase in oxygen metabolism (Liao et al., 2013; Scholkmann et al., 2014; Pinti et al., 2020), leading to a decrease and increase in the concentration of oxygenated hemoglobin and deoxygenated hemoglobin (Lindauer et al., 2010; Liao et al., 2013; Scholkmann et al., 2014). The results of fNIRS has higher spatial resolution than portable electroencephalography, and has the highest correlation to functional magnetic resonance imaging BOLD measures (Strangman et al., 2002). In addition, fNIRS has been used to detect the cortical activity of the prefrontal cortex (PFC), primary cortex (M1), pre-motor cortex (PMC), supplementary motor area (SMA), and sensory-motor cortex (SMC) of the healthy or unhealthy population under single or dual-tasks during walking, turning, or balance intervention (Mihara et al., 2007; Al-Yahya et al., 2018; Stuart et al., 2018; Pelicioni et al., 2022), while portable electroencephalography based on neuroelectric signals of neurons is rarely used in dual‐task gait activities compared with fNIRS. PFC takes part in planning, regulating and controlling of movement mainly (Szczepanski and Knight, 2014); SMC and M1 take part in planning, control and motor execution (Donoghue et al., 1994; Zwergal et al., 2012); SMA and PMC take part in planning and selecting movement (Gazzaniga, Ivry, and Mangun, 2009). Therefore, PFC, M1, PMC, SMA and SMC are all associated with movement.

Cortical activation and gait characteristics of PD patients (Stuart et al., 2019; Bonilauri et al., 2020), stroke patients (Chen et al., 2017; Herold et al., 2017), MS patients (Bonilauri et al., 2020), and cognitive impairment patients (Bishnoi et al., 2021) related to older neurological disorder patients with dyskinesia have been described under single or dual tasks during walking or balance intervention in former reviews touch on fNIRS. However, no review has tackled the relationship between motor performance and cortical activation of older neurological disorder patients while performing motor tasks. Therefore, this study aims to review the relationship between motor performance and cortical activity of older PD, stroke, and MS patients during walking and balance tasks. It might help improve patients’ rehabilitation for intervention development to be uncovered.

## 2 Methods

### 2.1 Search strategy

**FIGURE 1:**
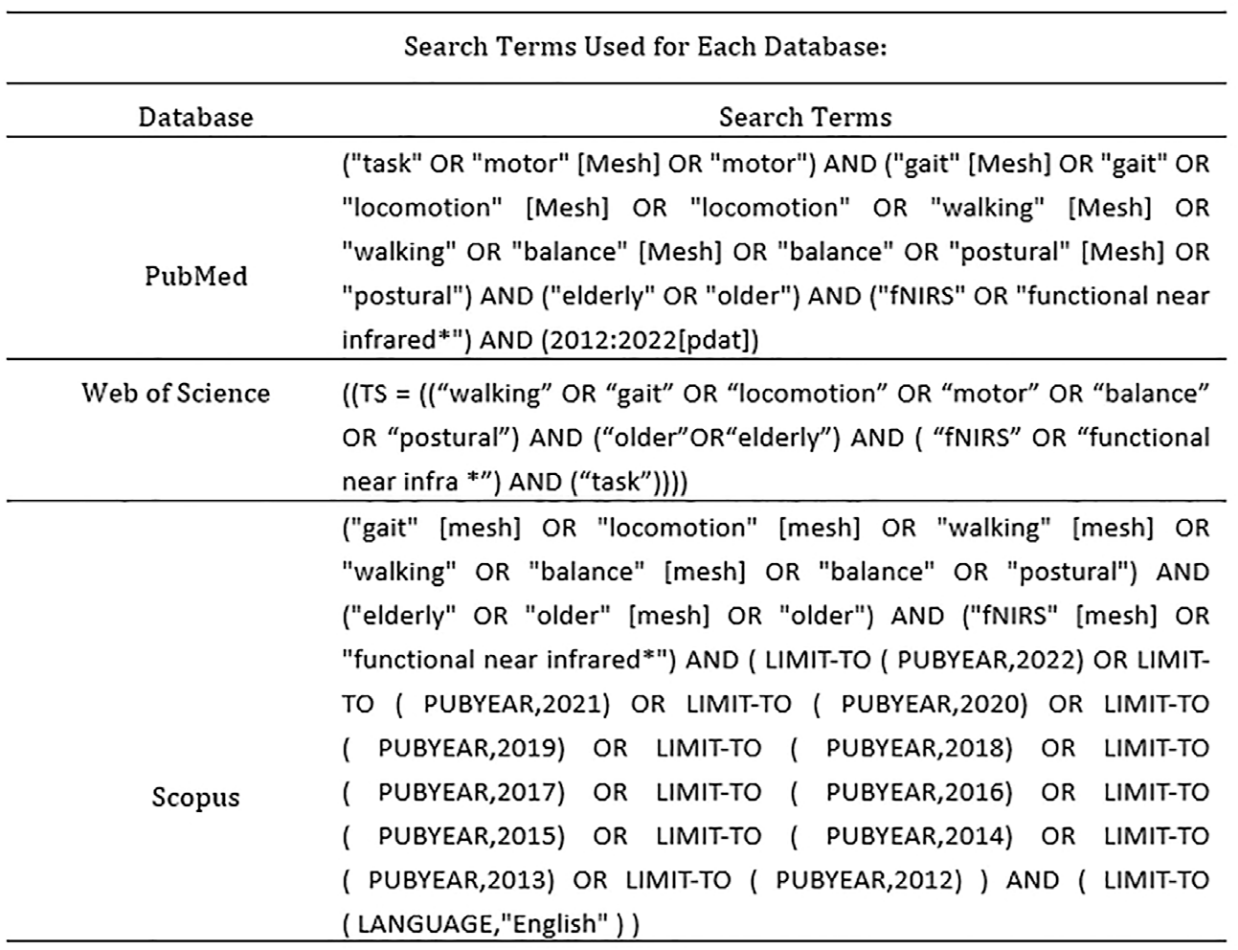
Search string key terms used for study acquisition.

Two independent researchers performed a systematic literature search in Scopus, PubMed, and Web of Science databases to identify all relevant studies published from 1 January 2012 to 30 December 2022 after the fNIRS became popular (Menant et al., 2020; Pinti et al., 2020). The 2020 Preferred Reporting Items for Systematic Reviews and Meta-Analyses (PRISMA) statements were used to report this systematic review. Key search terms and synonyms of three database are shown in Figure 1.

### 2.2 Eligibility criteria

**FIGURE 2:**
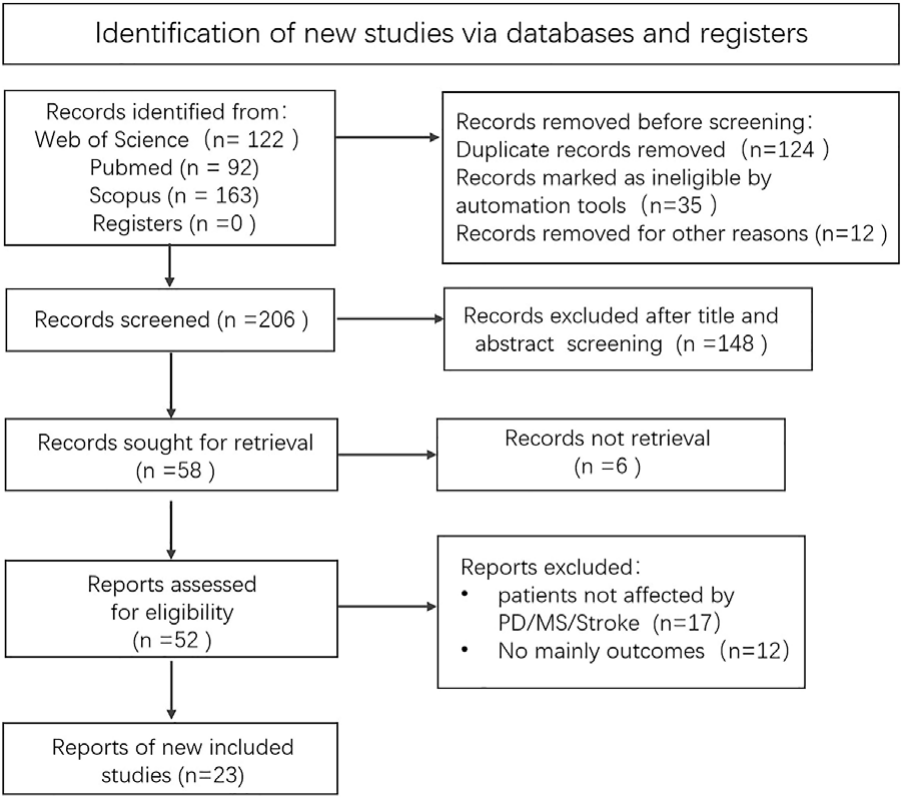
Search and screening process.

Search and screening process is shown in Figure 2. Herein, studies were included based on the following criteria: 1) the sample or subgroup included age 60 years or older adults (Rudnicka et al., 2020) with neurological diseases such as PD, MS, and stroke, 2) fNIRS was used as the cortical activation test equipment, 3) gait or balance performance with kinematic results or score, and cortical activity were the primary result, 4) articles were written in English.

However, studies were deemed irrelevant based on the following criteria: 1) it was non-experimental studies (i.e., review and empirical studies), 2) participants were healthy people only, people with other diseases or animal, 3) other cortical activation test equipment (i.e., EEG), was used, 4) main results were not kinematics and cortical activation, 5) the full text of study was not available, 6) a brain-computer interface was used, 7) studies were the conferences, or case reports.

### 2.3 Study selection

A stepwise process was used to identify articles to be selected for inclusion in this review. Two authors independently screened titles, followed by abstracts, and then full texts. Reviewers (YW, YD, WW, and YB) extracted the data and synthesized it into pre-designed tables. If any disagreement arose regarding the inclusion or exclusion of single studies, it would be resolved through consultation and confirmed by the reviewers (ZC and YT).

### 2.4 Data extract process

The following items were extracted from each included study; the name of author and published year; the type of neurological disorders (i.e., PD, stroke), age and degree of dyskinesia (i.e., freezing of gait); the number of fNIRS channels and region of interest; type of motor task and task paradigms (i.e., single task, dual task); intervention time; motor performance and cortical activation results including statistical correlation results, gait characteristics, balance score, and HbO_2_ level of region of interest.

### 2.5 Risk of bias in individual studies

Each full-text article was assessed by two independent reviewers and scored using the PEDro scale. Any discrepancies were resolved by discussion and consensus. The PEDro scale was developed to measure methodological quality and internal validity of randomized studies (de Morton, 2009). Each of the 10 items is scored as either presenter absent (0) and a score is then calculated out of 10 with a larger number indicating better quality (Mason et al., 2016). The categories that were used to define overall quality of each article are as follows: ≦ 4 (poor), 5–6 (moderate) and ≧ 7 (high quality) (Fernandez et al., 2016).

## 3 Results

### 3.1 Overview of included studies

As shown in Figure 2, a total of 377 studies were identified through key terms search, 206 studies were left in removed duplicates and so on, 148 were excluded in the title and abstract screening, 52 studies were reviewed in full texts, and 29 of them were excluded for not meeting the mentioned selection criteria. The remaining 23 studies were included in this review.

Tables 1–3 showed the 23 studies included in this study. Of these, studies assessed patients with PD (N = 13), with a previous history of stroke (N = 8), and with MS (N = 2). The age of older patients in these studies was widely distributed from 56 ± 5 to 73 ± 1 year. Studies contain cross-sectional (N = 19) and longitudinal designs (N = 4).

**TABLE 1:** Summary of motor performance and cortical activation outcomes in studies of Parkinson’s patients.

Authors	Participant characteristics	fNIRS	Task paradigms	Motor performance and cortical activation results
Year		ROIs		
Maidan et al. (2015)	11 PD (66.2 ± 10.0), FOG history	Channels (not mentioned)	ST1: normal walking	↓The turning acceleration, ↑HbO_2_ level in PFC in FOG shows under ST2 condition compared to without FOG shows.
	11 HOA (71.2 ± 6.0)	PFC	ST2: 180° turn	
Maidan et al. (2016)	68 PD (71.7 ± 1.1)	6 channels	ST: normal walking	A positive correlation between HbO_2_ level (↑) and step speed (↑) during obstacle negotiation walking. (*)
	38 HOA (70.4 ± 0.9)	PFC	DT1: ST + n-back	
			DT2: ST + obstacles negotiation	
Mahoney et al. (2016)	269 non-demented adults (76.41 ± 6.70):	16 channels	stand upright and count silently for 10 s	↑COP velocity, ↑ HbO_2_ level in PFC in PS compared with HOA.
	117 MPS	PFC		
	26 PS			
	126 HOA			
Maidan et al. (2017)	49 PD (71.7 ± 1.0)	3 channels	2 min walk test (straight line + 180° turns)	A negative correlation between PFC activation (↑) and step speed (↓) during turning in the PD group. (*)
	126 HOA	PFC		
Al-Yahya et al. (2018)	29 PD (66.3 ± 5.9)	6 channels	ST: self-selected walking speed and fast walking speed	←Step characteristics, ↑HbO_2_ level in PFC and M1 during DT condition compared with ST condition in PD group.
	22 HOA (59.5 ± 6.8)	PFC, M1	DT: ST + n-back	
Maidan et al. (2018)	64 PD:	6 channels	ST1: normal walking	↑Step speed and step length, ↓HbO_2_ level in PFC during walking after treadmill training.
	34 TT (73.1 ± 1.1)	PFC	DT1: ST + n-back	
	30 TT + VR (70.1 ± 1.3)		DT2: ST + obstacles negotiation	
			6 weeks of treadmill training.	
Thumm et al. (2018)	20 PD (69.8 ± 6.5)	Channels (not mentioned)	ST1: overground walking	↓Step speed, ↓Hb0_2_ level in PFC during ST2 condition compared to ST1 condition.
		PFC	ST2: treadmill walking	
Belluscio et al. (2019)	15 PD + FOG (66.9 ± 5.0)	8 channels	ST: 360° turns in place	A negative correlation between PFC activation (↑) and turning performance (↓) (↓number of turns in PD patients without FOG in DT during turning). (*)
	17PD + nonFOG (69.9 ± 4.3)	PFC	DT: ST + auditory AX-continuous	↓Step speed, ↓HbO_2_ level in PFC for FOG during DT compared with ST.
	8 HOA (66.5 ± 5.5)			↓Step speed, ↑HbO_2_ level during turning compared with HOA.
Stuart and Mancini (2020)	13 PD FOG (69.7 ± 4.2)	2 channels	ST1: 180° and 360° turns while walking	↓Step speed, ←PFC activity during walking or turning under DT condition compared with ST2 condition in the PD group.
	12 PD nonFOG (68.7 ± 3.9)	PFC	ST2: walking back and forth	
			DT: ST2+ auditoryAX-continuous with an open-or closed-loop tactile cue	
Vitorio et al. (2020)	24 PD + FoG (70.3 ± 4.7)	8 channels	ST: walking back and forth	↓Step speed and step length, ↓HbO_2_ level in PFC under DT condition compared with ST condition in FOG group.
	23PD + nonFOG (70.8 ± 7.6)	PFC	DT: ST + auditoryAX-continuous	↑Step time variability, ↑HbO_2_ level in PFC during walking in FOG group compared with nonFOG group.
Orcioli-Silva et al. (2021)	36 PD: 17 TD (70.8 ± 6.8) 19 PIGD (69.4 ± 6.6)	8 channels	ST: normal walking	↓Step speed and step length, ↑HbO_2_ level in PFC during DT condition compared with ST condition.
		PFC	DT: obstacle negotiation walking	
Hoang et al. (2022)	14 PD (67 ± 9)	8 channels DLPFC	ST: walking	A positive correlation between ΔHbO_2_ of DLPFC (↓) and coefficient of variation of step length (↓). (*) ↑Step speed and step length, ↓HbO_2_ level in DLPFC during T2 compared with T1.
			5 weeks of Sirocco training	
			T0 and T1: before training	
			T2: after training	
Pelicioni et al. (2022)	49 PD (69.5 ± 7.9)	6 channels	ST1: simple 60 s-walking trial	↓Step length and step speed, ↑PMC activation in PD group compared with HOA. ↓Step length and step speed, ↑SMA activation in ST2 condition compared with ST1 condition in PD group.
			ST2: obstacle negotiation	
	21 HOA (69.0 ± 5.9)	DLPFC, SMA, PMC	ST3: short and long target stepping	
			DT: ST1+ST2+ST3	

Abbreviations: HOA = healthy older adult, PD = Parkinson’s disease, FOG = freezing of gait, PS = Parkinsonian syndromes, MPS = mild parkinsonian signs, TD = tremor dominant, PIGD = postural instability step disorder, Age = Mean ± SD, fNIRS = functional near infrared spectroscopy, PFC = prefrontal cortex, DLPFC = Dorsolateral prefrontal cortex, SMA = supplementary motor area, PMC = premotor cortex, ST = single-task, DT = dual-task, TT = treadmill training, VR = virtual reality, ↑:increase significantly, ↓:decrease significantly, ←: no significant differences, (*): Statistical correlation.

**TABLE 2:** Summary of motor performance and cortical activation outcomes in studies of stroke patients.

Authors	Participant characteristics	fNIRS	Task paradigms	Motor performance and cortical activation results
Year		ROIs		
Mihara et al. (2012)	20 Stroke (61.9 ± 11.9)	50 channels	balance control task	A positive correlation between BBS score (↑) and the changes in HbO_2_ (↑). (*)
		PFC, SMA		
Fujimoto et al. (2014)	20 Stroke (60.2 ± 9.5)	50 channels	balance control task	↑BBS score (postural stability) and ↑HbO_2_ level in bilateral SMA after the intervention compared with before the intervention.
		PFC, SMA	about 6 weeks	
Mori et al. (2018)	14 Stroke (61.1 ± 9.3)	16 channels	ST: walking around a circle	↓Step speed and ↓HbO_2_ level in PFC under DT condition in stroke group compared with HOA group.
	14 HOA (66.3 ± 13.3)	PFC	DT: ST + n-back	
Hawkins et al. (2018)	24 Stroke (58 ± 9.3)	4 channels	ST: normal walking	↓Step speed and ↑HbO_2_ level in PFC under DT2 condition in stroke group compared with HOA group.
	15 HOA (77.2 ± 5.6)	PFC	DT1: ST + verbal letter fluency	
			DT2: ST + obstacles negotiation	
Caliandro et al. (2020)	22 Stroke (60 ± 8.1)	2 channels	walking with and without the wearable powered exoskeletons	A positive correlation between Knutsson score (↑) (abnormal muscle activation) of non-paretic limb and HbO_2_ level in PFC (↑) during walking. (*)
	15 HOA (43–69)	PFC		
Collett et al. (2021)	45 Stroke (62 ± 14): 21 Good walker	32 channels	ST: normal walking	↓Step speed and step length, and↑HbO_2_ level in PFC under DT condition, compare ST.
	24 Limited walker	PFC	DT: ST + stroop treadmill walking for approximately 10 weeks	
Lim et al. (2022a)	20 Stroke (64 ± 7.6)	54 channels	ST: normal walking	↓Step speed and ↑HbO_2_ level in PFC, SMC, and PMC under DT condition compared with ST condition.
		PFC, SMC, PMC	DT: ST + verbal letter fluency	
Lim et al. (2022b)	20 Stroke (64 ± 7.6)	54 channels	normal walking	A positive correlation between step speed (↑) and cortical activity of PFC, SMC, and PMC (↑) of stroke under the acceleration stage of walking. (*)
		PFC, SMC, PMC		

Abbreviations: HOA = healthy older adult, Age = Mean ± SD, fNIRS = functional near infrared spectroscopy, PFC = prefrontal cortex, SMC = sensorimotor cortex, SMA = supplementary motor area, PMC = premotor cortex, ST = single-task., DT = dual-task, Berg Balance Scale = BBS, ↑: increase significantly, ↓: decrease significantly, (*): Statistical correlation.

**TABLE 3:** Summary of motor performance and cortical activation outcomes in studies of MS patients.

Authors	Participant characteristics	fNIRS	Task paradigms	Motor performance and cortical activation results
Year		ROIs		
Hernandez et al. (2016)	8 MS (61 ± 4)	2 channels	ST: normal walking	↓Step speed and ↑HbO_2_ level in PFC under DT condition compared with HOA or ST condition in MS group.
	8 HOA (57 ± 5)	PFC	DT: ST + reciting alternate letters	
Chaparro et al. (2017)	10 MS (56.2 ± 5.1)	16 channels	ST: walking on a treadmill with no or partial body weight support	↓Step speed and ↑HbO_2_ level in PFC during walking compared with HOA.
	12 HOA (63.1 ± 4.4)	PFC	DT: ST + reciting alternate letters	

Abbreviations: HOA = healthy older adult, MS = multiple sclerosis, Age = Mean ± SD, fNIRS = functional near infrared spectroscopy, PFC = prefrontal cortex, ST = single-task, DT = dual-task, ↑: increase significantly, ↓: decrease significantly, (*): Statistical correlation.

Among the 23 studies, walking was used as a test task in 20 studies, including straight walking (N = 15), turning (N = 5), obstacles (N = 5), and treadmill walking (N = 4). Meanwhile, the balance was used as a test task in the other three studies, including normal standing (N = 1) and postural intervention standing (N = 2). Gait and posture stability were the primary behavioral outcomes of walking and balance tasks. PFC was the most popular region of interest (N = 19) which was tested by 2–32 channels in fNIRS systems. Other region of interest in the frontal and parietal cortices were tested by six channels (N = 2) or more than 40 channels in the fNIRS system (N = 4).

### 3.2 Risk of bias in individual studies

Scores on the PEDro scale ranged from five to eight for the selected articles (Table 4). Twenty one studies were of moderate quality; and two studies were high quality.

**TABLE 4:** Hysiotherapy evidence database (PEDro) Scale scores of the reviewed studies.

PEDro criteria	1	2	3	4	5	6	7	8	9	10	Total
Maidan et al. (2015)	Yes	No	No	Yes	No	No	Yes	Yes	Yes	Yes	6
Maidan et al. (2016)	Yes	No	No	Yes	No	No	No	Yes	Yes	Yes	5
Mahoney et al. (2016)	Yes	No	No	Yes	No	No	Yes	Yes	Yes	Yes	6
Maidan et al. (2017)	Yes	No	Yes	Yes	No	No	No	Yes	Yes	Yes	6
Al-Yahya et al. (2018)	Yes	No	No	Yes	No	No	No	Yes	Yes	Yes	5
Maidan et al. (2018)	Yes	Yes	Yes	Yes	No	No	Yes	Yes	Yes	Yes	8
Thumm et al. (2018)	Yes	No	No	Yes	No	No	No	Yes	Yes	Yes	5
Belluscio et al. (2019)	Yes	No	No	Yes	No	No	No	Yes	Yes	Yes	5
Stuart and Mancini (2020)	Yes	No	No	Yes	No	No	No	Yes	Yes	Yes	5
Vitorio et al. (2020)	Yes	No	No	Yes	No	No	No	Yes	Yes	Yes	5
Orcioli-Silva et al. (2021)	Yes	No	No	Yes	No	No	No	Yes	Yes	Yes	5
Hoang et al. (2022)	Yes	No	No	Yes	No	No	No	Yes	Yes	Yes	5
Pelicioni et al. (2022)	Yes	Yes	No	Yes	No	No	No	Yes	Yes	Yes	5
Mihara et al. (2012)	Yes	Yes	No	Yes	No	No	No	Yes	No	Yes	5
Fujimoto et al. (2014)	Yes	Yes	No	Yes	No	No	No	Yes	No	Yes	5
Mori et al. (2018)	Yes	No	No	Yes	No	No	No	Yes	Yes	Yes	5
Hawkins et al. (2018)	Yes	No	No	Yes	No	No	No	Yes	Yes	Yes	5
Caliandro et al. (2020)	Yes	No	No	Yes	No	No	No	Yes	Yes	Yes	5
Collett et al. (2021)	Yes	No	Yes	Yes	Yes	No	No	Yes	Yes	Yes	7
Lim et al. (2022a)	Yes	No	No	Yes	No	No	No	Yes	Yes	Yes	5
Lim et al. (2022b)	Yes	Yes	No	Yes	No	No	No	Yes	No	Yes	5
Hernandez et al. (2016)	Yes	No	No	Yes	No	No	Yes	Yes	Yes	Yes	6
Chaparro et al. (2017)	Yes	No	No	Yes	No	No	Yes	Yes	Yes	Yes	6

Criteira: 1) Eligibility; 2) Randomized allocation; 3) Concealed allocation; 4) Baseline similarity; 5) Blinded subjects; 6) Blinded therapists; 7) Blinded assessors; 8) Key outcomes; 9) Comparison between groups; 10) Point measures and measures of variability.

### 3.3 Co-direction change of motor performance and cortical activation

Seven studies reported increased gait or balance results and increased cortical activation. One of them found a positive correlation between step speed and cortical activation of PFC during obstacle negotiation walking of PD patients *via* Pearson’s correlation methods (Maidan et al., 2016). Three of them found a positive correlation between Berg Balance Scale, and cortical activation of PFC and SMA of stroke patients *via* Spearman’s correlation methods (Mihara et al., 2012), Knutsson score (abnormal muscle activation) and cortical activation of PFC of stroke patients with exoskeletons by multiple regression methods (Caliandro et al., 2020); and step speed and cortical activity of PFC, SMC, and PMC of stroke patients *via* Pearson’s correlation methods (Lim et al., 2022b). Three studies found that Center of Pressure velocity and cortical activation of PFC were increased (Mahoney et al., 2016); step time variability and cortical activation of PFC of PD patients with freezing of gait compared with non-freezing of gait during walking were increased (Vitorio et al., 2020); BBS score and cortical activation of SMA were increased during balance task after neuro-modulative therapies rehabilitation (Fujimoto et al., 2014).

Five studies reported decreased gait or balance results and decreased cortical activation. One of them found a positive correlation between coefficient of variation of step length, and cortical activation of dorsolateral prefrontal cortex of PD patients *via* Pearson’s correlation methods (Hoang et al., 2022). Three of them found step speed and PFC cortical activation of PD patients were decreased during dual-task condition compared with single-task condition (Belluscio et al., 2019; Vitorio et al., 2020), and during treadmill walking compared with over-ground walking (Thumm et al., 2018). One of them found step speed and cortical activation of PFC were decreased under dual-task condition of stroke patients compared with healthy older adults (Mori., 2018).

### 3.4 Inverse change of motor performance and cortical activity

Ten studies reported decreased gait or balance results and increased cortical activation. Two of them found negative correlations between increased PFC activation and decreased step speed (Maidan et al., 2017), and the decreased number of turns (Belluscio et al., 2019) of PD patients under dual-task conditions *via* Pearson’s correlation methods. Two of them found that step speed was decreased (Belluscio et al., 2019; Pelicioni et al., 2022), cortical activation was increased in PFC (Belluscio et al., 2019) and PMC (Pelicioni et al., 2022) of PD patients compared with healthy participants. One of them found that turning acceleration was decreased during turning, and cortical activation of PFC was increased in PD patients with freezing of gait compared with non-freezing of gait (Maidan et al., 2015). Two of them found that step speed and length were decreased, cortical activation of PFC (Orcioli-Silva et al., 2021) and SMA (Pelicioni et al., 2022) were increased under obstacle negotiation dual-task conditions compared with single task condition. Three of them found step speed was decreased, cortical activation of PFC (Hawkins et al., 2018; Collett et al., 2021; Lim et al., 2022a), SMC (Lim et al., 2022a), and PMC (Lim et al., 2022a) were increased in stroke patients during walking under dual-task conditions compared with single-task condition (Collett et al., 2021; Lim et al., 2022a) or compared with healthy older adults (Hawkins et al., 2018). Two studies of MS patients found that step speed was decreased and cortical activation of PFC was increased under dual-task conditions (Hernandez et al., 2016) during walking compared to healthy older adults (Chaparro et al., 2017).

Two longitudinal studies reported step speed and length were increased, cortical activation of PFC or dorsolateral PFC was decreased after intervention (Maidan et al., 2018; Hoang et al., 2022).

One study reported gait characteristics did not change while cortical activation of PFC and M1 were increased of PD patients under dual-task conditions compared with single task condition or healthy older adults (Al-Yahya et al., 2018); the other one study reported step speed was decreased while cortical activation of PFC did not change of PD patients under dual-task conditions compared with single-task condition (Stuart and Mancini, 2020).

## 4 Discussion

A total of 23 studies that investigated the motor performance and cortical activity *via* fNIRS were systematically reviewed in this study. It showed varied relationships between motor performance and cortical activity of older adults with PD, stroke, and MS patients under walking and balance tasks.

### 4.1 Poor performance with changed cortical activation

Among the 23 studies, it was found that step speed and length as well as the number of turns were decreased, and the Center of Pressure velocity, step time variability, Knutsson score were increased. Those results usually used to indicate poor motor performance (Mancini et al., 2017; Belluscio et al., 2019; Kahya et al., 2019; Caliandro et al., 2020). However, the cortical activation of PFC, PMC, SMC, and SMA was increased or decreased during different motor and balance task.

Poor motor performance accompanied by increased PFC, PMC, SMC, and SMA activation indicated that automatic walking function was affected by the disease (Herold et al., 2017; Maidan et al., 2017; Pelicioni et al., 2022), because walking was controlled by two pathways in the brain (la Fougère et al., 2010; Zwergal et al., 2012; Zwergal et al., 2013; Herold et al., 2017). The direct and normal pathways relevant to automatic movement are controlled by the striatum and activated in the case of low-challenging walking and the absence of pathology. The indirect pathway relevant to motor-related cognitive resources involving the PFC, PMC, and SMC areas is activated in the impaired automatic walking caused by aging or disease (Vandenbossche et al., 2012; Clark, 2015; Herold et al., 2017; Al-Yahya et al., 2018). SMA cortex was also involved in inter-limb coordination, gait, and postural control (Fujimoto et al., 2014), which might activate in the complex obstacle negotiation task.

Therefore, poor motor performance with increased cortical activation might indicate that automatic walking turns into voluntary cortical control walking (Maidan et al., 2015), which might be a cognition and postural compensation to counter the motor or neuron dysfunction of neurological disorder patients with dyskinesia.

However, poor motor performance accompanied by decreased PFC activation indicates that once the compensatory is over-activated, the cognitive resources would reach the limit (Holtzer and Izzetoglu, 2020). Dual-task and circle walking are both difficult tasks for neurological disorder patients with dyskinesia. The changes in brain structure and function caused by that disease might easily induce the inflexibility or overburdening of executive attention cognitive resources (Stuart and Mancini, 2020). Consequently, the neural efficiency of PFC becomes lower, the executive function goes down, the cortical activation of PFC reaches the ceiling, the impaired motor function cannot get support from the cortex, and the motor performance becomes poor.

### 4.2 Better performance with changed cortical activation

Among the 23 studies, it was found that step speed, BBS score and length were increased, and the coefficient of variation of step length and step speed were decreased. Those results usually used to indicate improved motor performance (Collett et al., 2021; Hoang et al., 2022), and improved balance control (Fujimoto et al., 2014). However, sometimes the cortical activation of PFC decreased, sometimes the cortical activation of PFC, SMA, PMC, and SMC increased during different motor task.

Better motor performance accompanied by decreased PFC activation showed a positive effect of sports training on neurological disorder patients with dyskinesia. It indicates that the automaticity of walking was improved, the dependence on cognitive resources was reduced, and the patients could make better use of their executive resources to walk normally (Hoang et al., 2022). It might be because sports training enhances neural plasticity to a certain extent, thereby promoting angiogenesis, neurogenesis, and synaptogenesis, all of which led to an increased efficiency and help reduce the required levels of cortex activation (Maidan et al., 2018).

Better motor performance accompanied by increased PFC, PMC, SMA, and SMC activation also showed positive effects on balance and normal walking tasks. Based on the compensatory reallocation model (Verghese et al., 2017), the brain recruits neural-related area and allocates more neural resources to the motor program when the task was slightly difficult for patients, thereby resulting in near-normal and safe motor performance finally. Therefore, higher cortical activation caused by balance and walking helped patients with limited motor ability to improve their motor performance.

Better motor performance accompanied by increased SMA activation showed a positive effect on balance task skills. SMA was related to inter-limb coordination and postural control (Fujimoto et al., 2014), and was a potential target of neuro-modulative therapies which can promote recovery of motor function (Hummel and Cohen, 2006). Therefore, the advanced SMA cortical activation would help recover the balance function of stroke patients.

### 4.3 Changed performance or cortical activation *versus* unchanged

Among the 23 studies, one study reported unchanged step character accompanied by increased cortical activation of PFC and M1, the other one reported decreased step speed accompanied by unchanged cortical activation of PFC under dual-task conditions during walking.

Based on the compensatory reallocation model, in some motor tasks, the brain recruits neural network tissue in the motor-related cortex and allocates more neural resources to the motor program, thereby resulting in near-normal motor performance and high cortical activation (Verghese et al., 2017). Therefore, the brain would regulate the cortex resources and help patients to complete difficult walking. Patients with limited motor ability can maintain motor performance through improved cortical activation. However, if the resources of PFC are occupied by the additional task and reach the ceiling, it might be hard for patients to maintain motor performance well (Holtzer and Izzetoglu, 2020).

### 4.4 Learning from the change of relationships

Human movement is executed by the muscles and controlled by the Nervous system (Clark et al., 2014). The muscle fibers receive the input information from alpha neurons. The alpha neurons receive the input information from spinal fibers and neurons. The spinal receives input from extra-pyramidal tracts. The extra-pyramidal tracts receive the input from the cortical (i.e., PFC, M1, PMC, and SMA) and subcortical structure (Gazzaniga et al., 2009). Therefore one of symptom of neurological disorder patient is dyskinesia. The dyskinesia is associated with the abnormal of motor control which showed abnormal cortical activation and motor performance.

With the development of the realtime human movement cortical activation test equipment, more and more studies follow the interest of abnormal motor control of dyskinesia neurological disorder patients. However, the design and results were not comprehensive and profound. Most of the reviewed studies reported poor motor performance and increased cortical activation of PD, stroke and MS older patients under simple motor task conditions, compare with healthy older adults or difficult motor task conditions. Few studies discussed the exact relationship between motor performance and cortical activation, or used statistical analysis. The external motor performance such as step speed and step length were analyzed only. The sports biomechanics variables (i.e., muscle activation, segment movement) and its relationship with cortical activation were left behind.

Our review results showed that motor performance has improved after sports training or occupational therapy intervention. However, the type of sports training was simple, the difficulty, intensity, time interval of sports training intervention needs further study. The expanded and specific study would promote the clinical rehabilitation intervention therapy of neurological disorder patient with dyskinesia.

## 5 Conclusion

From the 23 included publications, four were interventional studies, seven studies used statistical methods to interpret the relationship between motor performance and cortical activation, participants were mostly PD patients, PFC was the favorite region of interest, and walking was the most popular test motor task. The motor performance and cortical activation of frontal and parietal cortices were simultaneously affected during difficult walking and balance tasks of the neurological disorder patients with dyskinesia. Most reviewed studies reported poor motor performance and increased cortical activation of PD, stroke and MS older patients, compare with healthy older adults or difficult motor task conditions. Few studies discussed the exact relationship of motor performance and cortical activation by statistical analysis. The design and results were not comprehensive and profound. The sports biomechanics variables and its relationship with cortical activation variables were left behind. More than 5 weeks of walking training or physiotherapy can contribute to motor function promotion as well as frontal and parietal cortices activation of PD and stroke patients.

## 6 Limitation

There are several limitations in the present review. Firstly, it was limited to studies published in English. As such, studies with relevant findings may have been ommited. Secondly, the search time period for this review was the recent 10 years while fNIRS became popular. As such, we might not identify all relevant articles. Thirdly, it was limited to studies containing walking and balance motor tasks. As such, studies relevant to upper extremity motor tasks were excluded. Fourthly, the other cortical activation detected methods, such as portable electroencephalography were not included in this review, it is not known if a different detected method could have different results. Last but not least, the various study design made it difficult to summarize and discuss the different neural pathway changes of walking and balance control.

## 7 Future scope

Further study is needed to do more statistical analysis on the relationship between motor performance and activation of motor-related cortex. It would help analyze their relationship accurately. And dual task walking, obstacle walking and turning might be used as a sports training method. Intervention studies should be conducted to distinguish the intervention time, intensity and effects of dual-task walking, obstacle walking, over ground walking, and treadmill walking for neurological disorder patients with dyskinesia. It would help to look for new sport rehabilitation methods for those patients. Meanwhile, body performance such as trunk or head movement, electromyography, and ground reaction force should be synchronized with cortical activation test to evaluate the posture control of patients during walking and balance task. It would help to investigate the common change in musculoskeletal and nervous system, then further discuss their collaboration. Finally, the relationship between the different neural pathways of movement and the corresponding motor performance should be considered. It would help to investigate the different coordinate mechanisms of the musculoskeletal and nervous system in diverse patients movements.

## Data Availability

The original contributions presented in the study are included in the article/supplementary material, further inquiries can be directed to the corresponding author.

## References

[B1] Al-Yahya E. Mahmoud W. Meester D. Esser P. Dawes H. (2018). Neural substrates of cognitive motor interference during walking; peripheral and central mechanisms. Front. Hum. Neurosci. 12, 00536. 10.3389/fnhum.2018.00536 PMC6333849 30687049

[B2] Bacellar A. Pedreira B. B. Costa G. Assis T. (2017). Frequency, associated features, and burden of neurological disorders in older adult inpatients in Brazil: A retrospective cross-sectional study. BMC Health Serv. Res. 17, 504. 10.1186/s12913-017-2260-x 28738866 PMC5523147

[B3] Belluscio V. Stuart S. Bergamini E. Vannozzi G. Mancini M. (2019). The association between prefrontal cortex activity and turning behavior in people with and without freezing of gait. Neuroscience 416, 168–176. 10.1016/j.neuroscience.2019.07.024 31330231 PMC7778469

[B4] Bishnoi A. Holtzer R. Hernandez M. E. (2021). Brain activation changes while walking in adults with and without neurological disease: Systematic review and meta-analysis of functional near-infrared spectroscopy studies. Brain Sci. 11, 291. 10.3390/brainsci11030291 33652706 PMC7996848

[B5] Bonilauri A. Sangiuliano Intra F. Pugnetti L. Baselli G. Baglio F. (2020). A systematic review of cerebral functional near-infrared spectroscopy in chronic neurological diseases-actual applications and future perspectives. Diagn. (Basel) 10, 581. 10.3390/diagnostics10080581 PMC7459924 32806516

[B6] Caliandro P. Molteni F. Simbolotti C. Guanziroli E. Iacovelli C. Reale G. (2020). Exoskeleton-assisted gait in chronic stroke: An EMG and functional near-infrared spectroscopy study of muscle activation patterns and prefrontal cortex activity. Clin. Neurophysiol. 131, 1775–1781. 10.1016/j.clinph.2020.04.158 32506008

[B7] Chaparro G. Balto J. M. Sandroff B. M. Holtzer R. Izzetoglu M. Motl R. W. (2017). Frontal brain activation changes due to dual-tasking under partial body weight support conditions in older adults with multiple sclerosis. J. Neuroeng Rehabil. 14, 65. 10.1186/s12984-017-0280-8 28662727 PMC5493004

[B8] Chen M. Pillemer S. England S. Izzetoglu M. Mahoney J. R. Holtzer R. (2017). Neural correlates of obstacle negotiation in older adults: An fNIRS study. Gait Posture 58, 130–135. 10.1016/j.gaitpost.2017.07.043 28778021 PMC5645241

[B9] Chisholm A. E. Makepeace S. Inness E. L. Perry S. D. McIlroy W. E. Mansfield A. (2014). Spatial-temporal gait variability poststroke: Variations in measurement and implications for measuring change. Arch. Phys. Med. Rehabil. 95, 1335–1341. 10.1016/j.apmr.2014.02.014 24582619

[B10] Clark D. J. (2015). Automaticity of walking: Functional significance, mechanisms, measurement and rehabilitation strategies. Front. Hum. Neurosci. 9, 246. 10.3389/fnhum.2015.00246 25999838 PMC4419715

[B11] Clark D. J. Rose D. K. Ring S. A. Porges E. C. (2014). Utilization of central nervous system resources for preparation and performance of complex walking tasks in older adults. Front. Aging Neurosci. 6, 217. 10.3389/fnagi.2014.00217 25202270 PMC4142860

[B12] Collett J. Fleming M. K. Meester D. Al-Yahya E. Wade D. T. Dennis A. (2021). Dual-task walking and automaticity after Stroke: Insights from a secondary analysis and imaging sub-study of a randomised controlled trial. Clin. Rehabil. 35, 1599–1610. 10.1177/02692155211017360 34053250 PMC8524683

[B13] de Morton N. A. (2009). The PEDro scale is a valid measure of the methodological quality of clinical trials: A demographic study. Aust. J. Physiother. 55, 129–133. 10.1016/s0004-9514(09)70043-1 19463084

[B14] Defebvre L. Krystkowiak P. (2016). Movement disorders and stroke. Rev. Neurol. Paris. 172, 483–487. 10.1016/j.neurol.2016.07.006 27476417

[B15] Donoghue J. P. Sanes J. N. (1994). Motor areas of the cerebral cortex. J. Clin. Neurophysiol. 11, 382–396. 7962487

[B16] Fernandez M. Ferreira M. L. Refshauge K. M. Hartvigsen J. Silva I. R. Maher C. G. (2016). Surgery or physical activity in the management of sciatica: A systematic review and meta-analysis. Eur. Spine J. 25, 3495–3512. 10.1007/s00586-015-4148-y 26210309

[B17] Fougère C. L. Zwergal A. Rominger A. Förster S. Fesl G. Dieterich M. (2010). Real versus imagined locomotion: A [18F]-FDG PET-fMRI comparison. Neuroimage 50, 1589–1598. 10.1016/j.neuroimage.2009.12.060 20034578

[B18] Fujimoto H. Mihara M. Hattori N. Hatakenaka M. Kawano T. Yagura H. (2014). Cortical changes underlying balance recovery in patients with hemiplegic stroke. Neuroimage 85, 547–554. 10.1016/j.neuroimage.2013.05.014 23684871

[B19] Gazzaniga M. S. Ivry R. B. Mangun G. R. (2009). Cognitive neuroscience: The biology of the mind. United States: W. W. Norton.

[B20] Harmon B. Wells M. Park D. Gao J. (2019). Ultrasound elastography in neuromuscular and movement disorders. Clin. Imaging 53, 35–42. 10.1016/j.clinimag.2018.10.008 30308432

[B21] Hausdorff J. M. Lowenthal J. Herman T. Gruendlinger L. Peretz C. Giladi N. (2007). Rhythmic auditory stimulation modulates gait variability in Parkinson's disease. Eur. J. Neurosci. 26, 2369–2375. 10.1111/j.1460-9568.2007.05810.x 17953624

[B22] Hawkins K. A. Fox E. J. Daly J. J. Rose D. K. Christou E. A. McGuirk T. E. (2018). Prefrontal over-activation during walking in people with mobility deficits: Interpretation and functional implications. Hum. Mov. Sci. 59, 46–55. 10.1016/j.humov.2018.03.010 29604488 PMC5988641

[B23] Hernandez M. E. Holtzer R. Chaparro G. Jean K. Balto J. M. Sandroff B. M. (2016). Brain activation changes during locomotion in middle-aged to older adults with multiple sclerosis. J. Neurol. Sci. 370, 277–283. 10.1016/j.jns.2016.10.002 27772776

[B24] Herold F. Wiegel P. Scholkmann F. Thiers A. Hamacher D. Schega L. (2017). Functional near-infrared spectroscopy in movement science: A systematic review on cortical activity in postural and walking tasks. Neurophotonics 4, 041403. 10.1117/1.NPh.4.4.041403 28924563 PMC5538329

[B25] Hoang I. Ranchet M. Cheminon M. Derollepot R. Devos H. Perrey S. (2022). An intensive exercise-based training program reduces prefrontal activity during usual walking in patients with Parkinson's disease. Clin. Park Relat. Disord. 6, 100128. 10.1016/j.prdoa.2021.100128 34988428 PMC8704467

[B26] Holtzer R. Izzetoglu M. (2020). Mild cognitive impairments attenuate prefrontal cortex activations during walking in older adults. Brain Sci. 10, 415. 10.3390/brainsci10070415 32630216 PMC7407944

[B27] Hummel F. C. Cohen L. G. (2006). Non-invasive brain stimulation: A new strategy to improve neurorehabilitation after stroke? Lancet Neurol. 5, 708–712. 10.1016/s1474-4422(06)70525-7 16857577

[B28] Kahya M. Moon S. Ranchet M. Vukas R. R. Lyons K. E. Pahwa R. (2019). Brain activity during dual task gait and balance in aging and age-related neurodegenerative conditions: A systematic review. Exp. Gerontol. 128, 110756. 10.1016/j.exger.2019.110756 31648005 PMC6876748

[B29] Leff D. R. Orihuela-Espina F. Elwell C. E. Athanasiou T. Delpy D. T. Darzi A. W. (2011). Assessment of the cerebral cortex during motor task behaviours in adults: A systematic review of functional near infrared spectroscopy (fNIRS) studies. Neuroimage 54, 2922–2936. 10.1016/j.neuroimage.2010.10.058 21029781

[B30] Liao L. D. Tsytsarev V. Delgado-Martínez I. Li M. L. Erzurumlu R. Vipin A. (2013). Neurovascular coupling: *In vivo* optical techniques for functional brain imaging. Biomed. Eng. Online 12, 38. 10.1186/1475-925x-12-38 23631798 PMC3655834

[B31] Lim S. B. Peters S. Yang C. L. Boyd L. A. Liu-Ambrose T. Eng J. J. (2022a). Frontal, sensorimotor, and posterior parietal regions are involved in dual-task walking after stroke. Front. Neurol. 13, 904145. 10.3389/fneur.2022.904145 35812105 PMC9256933

[B32] Lim S. B. Yang C. L. Peters S. Liu-Ambrose T. Boyd L. A. Eng J. J. (2022b). Phase-dependent brain activation of the frontal and parietal regions during walking after stroke - an fNIRS study. Front. Neurol. 13, 904722. 10.3389/fneur.2022.904722 35928123 PMC9343616

[B33] Lindauer U. Dirnagl U. Füchtemeier M. Böttiger C. Offenhauser N. Leithner C. (2010). Pathophysiological interference with neurovascular coupling - when imaging based on hemoglobin might go blind. Front. Neuroenergetics 2, 25. 10.3389/fnene.2010.00025 20953238 PMC2955428

[B34] Mahoney J. R. Holtzer R. Izzetoglu M. Zemon V. Verghese J. Allali G. (2016). The role of prefrontal cortex during postural control in Parkinsonian syndromes a functional near-infrared spectroscopy study. Brain Res. 1633, 126–138. 10.1016/j.brainres.2015.10.053 26551767 PMC4860007

[B35] Maidan I. Bernad-Elazari H. Gazit E. Giladi N. Hausdorff J. M. Mirelman A. (2015). Changes in oxygenated hemoglobin link freezing of gait to frontal activation in patients with Parkinson disease: An fNIRS study of transient motor-cognitive failures. J. Neurol. 262, 899–908. 10.1007/s00415-015-7650-6 25636682

[B36] Maidan I. Bernad-Elazari H. Giladi N. Hausdorff J. M. Mirelman A. (2017). When is higher level cognitive control needed for locomotor tasks among patients with Parkinson's disease? Brain Topogr. 30, 531–538. 10.1007/s10548-017-0564-0 28439757

[B37] Maidan I. Nieuwhof F. Bernad-Elazari H. Bloem B. R. Giladi N. Hausdorff J. M. (2018). Evidence for differential effects of 2 forms of exercise on prefrontal plasticity during walking in Parkinson's disease. Neurorehabil Neural Repair 32, 200–208. 10.1177/1545968318763750 29546797

[B38] Maidan I. Nieuwhof F. Bernad-Elazari H. Reelick M. F. Bloem B. R. Giladi N. (2016). The role of the frontal lobe in complex walking among patients with Parkinson's disease and healthy older adults: An fNIRS study. Neurorehabil Neural Repair 30, 963–971. 10.1177/1545968316650426 27221042

[B39] Mancini M. Smulders K. Cohen R. G. Horak F. B. Giladi N. Nutt J. G. (2017). The clinical significance of freezing while turning in Parkinson's disease. Neuroscience 343, 222–228. 10.1016/j.neuroscience.2016.11.045 27956066 PMC5289743

[B40] Mason S. A. Morrison D. McConell G. K. Wadley G. D. (2016). Muscle redox signalling pathways in exercise. Role of antioxidants. Free Radic. Biol. Med. 98, 29–45. 10.1016/j.freeradbiomed.2016.02.022 26912034

[B41] Menant J. C. Maidan I. Alcock L. Al-Yahya E. Cerasa A. Clark D. J. (2020). A consensus guide to using functional near-infrared spectroscopy in posture and gait research. Gait Posture 82, 254–265. 10.1016/j.gaitpost.2020.09.012 32987345

[B42] Mihara M. Miyai I. Hatakenaka M. Kubota K. Sakoda S. (2007). Sustained prefrontal activation during ataxic gait: A compensatory mechanism for ataxic stroke? Neuroimage 37, 1338–1345. 10.1016/j.neuroimage.2007.06.014 17683949

[B43] Mihara M. Miyai I. Hattori N. Hatakenaka M. Yagura H. Kawano T. (2012). Cortical control of postural balance in patients with hemiplegic stroke. Neuroreport 23, 314–319. 10.1097/WNR.0b013e328351757b 22357394

[B44] Mori T. Takeuchi N. Izumi S. I. (2018). Prefrontal cortex activation during a dual task in patients with stroke. Gait Posture 59, 193–198. 10.1016/j.gaitpost.2017.09.032 29073516

[B45] Murray C. J. Atkinson C. Bhalla K. Birbeck G. Burstein R. Chou D. (2013). The state of US health, 1990-2010: Burden of diseases, injuries, and risk factors. Jama 310, 591–608. 10.1001/jama.2013.13805 23842577 PMC5436627

[B46] Murray C. J. Vos T. Lozano R. Naghavi M. Flaxman A. D. Michaud C. (2012). Disability-adjusted life years (DALYs) for 291 diseases and injuries in 21 regions, 1990-2010: A systematic analysis for the global burden of disease study 2010. Lancet 380, 2197–2223. 10.1016/s0140-6736(12)61689-4 23245608

[B47] Nutt J. G. Bloem B. R. Giladi N. Hallett M. Horak F. B. Nieuwboer A. (2011). Freezing of gait: Moving forward on a mysterious clinical phenomenon. Lancet Neurol. 10, 734–744. 10.1016/s1474-4422(11)70143-0 21777828 PMC7293393

[B48] Orcioli-Silva D. Vitório R. Beretta V. S. da Conceição N. R. Nóbrega-Sousa P. Oliveira A. S. (2021). Is cortical activation during walking different between Parkinson's disease motor subtypes? J. Gerontol. A Biol. Sci. Med. Sci. 76, 561–567. 10.1093/gerona/glaa174 32674140

[B49] Osoba M. Y. Rao A. K. Agrawal S. K. Lalwani A. K. (2019). Balance and gait in the elderly: A contemporary review. Laryngoscope Investig. Otolaryngol. 4, 143–153. 10.1002/lio2.252 PMC6383322 30828632

[B50] Pelicioni P. H. S. Lord S. R. Okubo Y. Menant J. C. (2022). Cortical activation during gait adaptability in people with Parkinson's disease. Gait Posture 91, 247–253. 10.1016/j.gaitpost.2021.10.038 34775227

[B51] Pinti P. Tachtsidis I. Hamilton A. Hirsch J. Aichelburg C. Gilbert S. (2020). The present and future use of functional near-infrared spectroscopy (fNIRS) for cognitive neuroscience. Ann. N. Y. Acad. Sci. 1464, 5–29. 10.1111/nyas.13948 30085354 PMC6367070

[B52] Reich S. G. Savitt J. M. (2019). Parkinson's disease. Med. Clin. North Am. 103, 337–350. 10.1016/j.mcna.2018.10.014 30704685

[B53] Rudnicka E. Napierała P. Podfigurna A. Męczekalski B. Smolarczyk R. Grymowicz M. (2020). The World Health Organization (WHO) approach to healthy ageing. Maturitas 139, 6–11. 10.1016/j.maturitas.2020.05.018 32747042 PMC7250103

[B54] Scholkmann F. Kleiser S. Metz A. J. Zimmermann R. Mata Pavia J. Wolf U. (2014). A review on continuous wave functional near-infrared spectroscopy and imaging instrumentation and methodology. Neuroimage 85, 6–27. 10.1016/j.neuroimage.2013.05.004 23684868

[B55] Socie M. J. Sosnoff J. J. (2013). Gait variability and multiple sclerosis. Mult. Scler. Int. 2013, 645197. 10.1155/2013/645197 23533759 PMC3603667

[B56] Strangman G. Culver J. P. Thompson J. H. Boas D. A. (2002). A quantitative comparison of simultaneous BOLD fMRI and NIRS recordings during functional brain activation. Neuroimage 17, 719–731. 12377147

[B57] Stuart S. Alcock L. Rochester L. Vitorio R. Pantall A. (2002). Monitoring multiple cortical regions during walking in young and older adults: Dual-task response and comparison challenges. Int. J. Psychophysiol. 135, 63–72. 10.1016/j.ijpsycho.2018.11.006 30471327

[B58] Stuart S. Mancini M. (2020). Prefrontal cortical activation with open and closed-loop tactile cueing when walking and turning in Parkinson disease: A pilot study. J. Neurol. Phys. Ther. 44, 121–131. 10.1097/npt.0000000000000286 31425309

[B59] Stuart S. Vitorio R. Morris R. Martini D. N. Fino P. C. Mancini M. (2018). Cortical activity during walking and balance tasks in older adults and in people with Parkinson's disease: A structured review. Maturitas 113, 53–72. 10.1016/j.maturitas.2018.04.011 29903649 PMC6448561

[B60] Szczepanski S. M. Knight R. T. (2014). Insights into human behavior from lesions to the prefrontal cortex. Neuron 83, 1002–1018. 10.1016/j.neuron.2014.08.011 25175878 PMC4156912

[B61] Thumm P. C. Maidan I. Brozgol M. Shustak S. Gazit E. Shema Shiratzki S. (2018). Treadmill walking reduces pre-frontal activation in patients with Parkinson's disease. Gait Posture 62, 384–387. 10.1016/j.gaitpost.2018.03.041 29626840

[B62] Vandenbossche J. Deroost N. Soetens E. Coomans D. Spildooren J. Vercruysse S. (2012). Freezing of gait in Parkinson's disease: Disturbances in automaticity and control. Front. Hum. Neurosci. 6, 356. 10.3389/fnhum.2012.00356 23335895 PMC3541536

[B63] Verghese J. Wang C. Ayers E. Izzetoglu M. Holtzer R. (2017). Brain activation in high-functioning older adults and falls: Prospective cohort study. Neurology 88, 191–197. 10.1212/wnl.0000000000003421 27927937 PMC5224713

[B64] Villringer A. Chance B. (1997). Non-invasive optical spectroscopy and imaging of human brain function. Trends Neurosci. 20, 435–442. 10.1016/s0166-2236(97)01132-6 9347608

[B65] Vitorio R. Stuart S. Mancini M. (2020). Executive control of walking in people with Parkinson's disease with freezing of gait. Neurorehabil Neural Repair 34, 1138–1149. 10.1177/1545968320969940 33155506 PMC7704905

[B66] Zwergal A. la Fougère C. Lorenzl S. Rominger A. Xiong G. Deutschenbaur L. (2013). Functional disturbance of the locomotor network in progressive supranuclear palsy. Neurology 80, 634–641. 10.1212/WNL.0b013e318281cc43 23345641

[B67] Zwergal A. Linn J. Xiong G. Brandt T. Strupp M. Jahn K. (2012). Aging of human supraspinal locomotor and postural control in fMRI. Neurobiol. Aging 33, 1073–1084. 10.1016/j.neurobiolaging.2010.09.022 21051105

